# Hashimoto’s thyroiditis presenting with cardiac tamponade: a case report

**DOI:** 10.11604/pamj.2023.46.62.41687

**Published:** 2023-10-18

**Authors:** Muhammadnur Rachim Enoch, Muhammad Irfan, Rachmad Budianto, Achmad Hardin

**Affiliations:** 1 Cardiology Department, Abdul Aziz Hospital in Singkawang, Kota Singkawang, Kalimantan Barat 79123, Indonesia

**Keywords:** Cardiac tamponade, Hashimoto’s thyroiditis, hypothyroidism, case report

## Abstract

Cardiac tamponade as the initial manifestation of Hashimoto's thyroiditis is an exceedingly uncommon occurrence. We present the case of a 36-year-old female who was admitted due to acute respiratory distress. A cardiac ultrasound revealed a severe pericardial effusion with tamponade signs. Subsequently, percutaneous pericardiocentesis was performed, resulting in a swift clinical improvement. Laboratory examinations confirmed severe hypothyroidism associated with Hashimoto's disease. Despite undergoing hormone replacement therapy, histological analysis of the pericardium revealed a chronic inflammation process. A follow-up cardiac ultrasound conducted six months later indicated the presence of a well-tolerated chronic pericardial effusion. In conclusion, clinicians should consider hypothyroidism as a potential cause when cardiac tamponade is observed, particularly in the absence of tachycardia. The prognosis is generally favorable with hormone replacement therapy, but regular ultrasound monitoring should be maintained until the patient achieves a euthyroid state.

## Introduction

Hashimoto's thyroiditis is an autoimmune disorder that affects the thyroid gland, an important producer of hormones regulating numerous bodily functions. This condition prompts the immune system to attack and destroy the thyroid's hormone-producing cells, typically resulting in decreased hormone production, a condition known as hypothyroidism. Hypothyroidism affects approximately 4% to 10% of the general population [1]. Insufficient thyroid hormones can impact nearly every organ system, leading to a variety of clinical symptoms. Thyroid hormones, particularly triodothyronine (T3), play a crucial role in development and function. Triodothyronine (T3) is responsible for increased tissue oxygen consumption, enhanced systolic contraction force, diastolic relaxation, and an overall reduction in vascular resistance. Hypothyroidism, characterized by a lack of T3, can manifest in cardiovascular issues such as diastolic hypertension, sinus bradycardia, pericarditis, dyslipidemia, and pericardial effusion [2].

Pericardial effusion is a condition characterized by the abnormal accumulation of fluid in the pericardial cavity and can result from various underlying causes such as tumors, uremia, trauma, cardiac surgery, and hypothyroidism. In hypothyroidism, the incidence of pericardial effusion is relatively low, occurring in approximately 3% to 6% of mild cases, and cardiac tamponade as the initial presentation of hypothyroidism is rare [3,4]. The progressive buildup of pericardial fluid can lead to cardiac tamponade. Typical acute cardiac tamponade is clinically diagnosed by observing symptoms like hypotension, jugular vein distention, and muffled heart sounds, collectively known as Beck's triad. In contrast, hypothyroid patients with Hashimoto's disease often present with bradycardia, a normal heart rate, or even high blood pressure [5,6].

In this article, we report a case from Abdul Aziz Hospital in which a patient with Hashimoto's disease developed cardiac tamponade, highlighting a rare occurrence associated with this condition.

## Patient and observation

**Patient information:** a 36-year-old female patient hailing from Singkawang City, Indonesia, received an initial diagnosis of primary hypothyroidism secondary to Hashimoto's thyroiditis. She sought medical attention due to a progressive increase in weight, worsening dyspnea, and lower leg edema. According to her statement, there is no history of similar complaints in her family.

**Clinical findings:** upon admission, the patient was in a state of respiratory distress, seated on her bed, with an oxygen saturation level below 85%, a respiratory rate of 35 breaths per minute, and a blood pressure measuring 84/55 mmHg. Clinical examination revealed a slight goiter upon palpation, without any signs of compression, decreased breath sounds, and pitted edema in both lower legs.

**Timeline:** the patient was admitted to the hospital on January 26, 2023, due to dyspnea, hypotension, and swelling in both legs, with a normal heart rate. Upon admission, the chest X-ray showed a “water bottle-shaped” image of the heart, and the echocardiography revealed the presence of pericardial fluid and a collapsed right ventricle. Emergency pericardiocentesis was performed, and 1000 ml of fluid was drained using a pigtail drain. One day later, an additional 500 ml of fluid was drained. Two days later, 500-800 ml of fluid was drained until the fifth day. On the fifth day, the patient no longer experienced dyspnea, and a repeat echocardiography showed minimal pericardial fluid. The patient was discharged with a prescription for 75 µg of levothyroxine tablet therapy. Six months later, the patient came to control with no complaints, a repeat echocardiography showed a well-tolerated chronic pericardial effusion and thyroid hormone was in the normal range.

**Figure 1:**
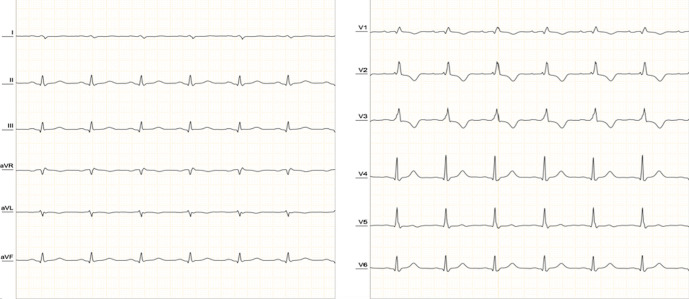
the ECG showing normal sinus rhythm with low voltage, right axis deviation (RAD), and incomplete right bundle branch block (RBBB)

**Figure 2:**
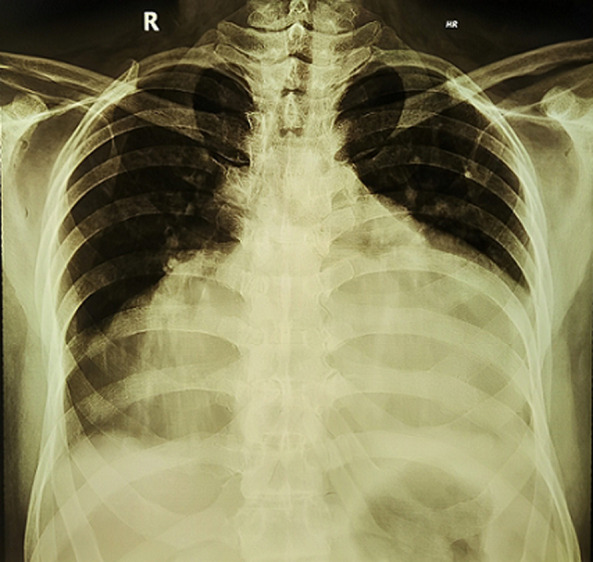
chest radiogram showing cardiomegaly with globular enlargement of the cardiac silhouette with “water bottle” configuration

**Figure 3:**
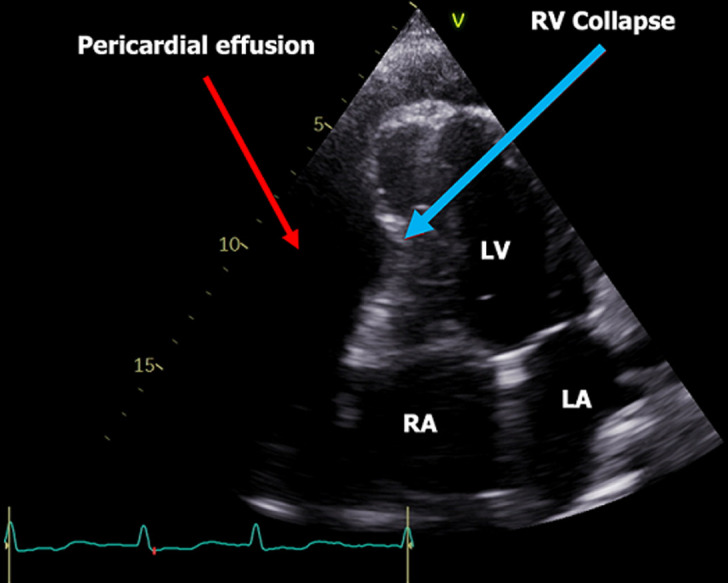
a two-dimensional transthoracic echocardiography reveals a significant pericardial effusion surrounding the heart; the red arrow indicates the effusion, while the blue arrow indicates right ventricular (RV) collapse; (LV: left ventricular, LA: left atrium, RA: right atrium)

**Diagnostic assessment:** an electrocardiogram (ECG) indicated low-voltage ECG complexes, right axis deviation, incomplete right bundle branch block, and sinus rhythm, with a heart rate of approximately 80 beats per minute (Figure 1). A chest X-ray displayed a characteristic “water bottle-shaped” sign, suggesting the presence of pericardial effusion (Figure 2). Further assessment through transthoracic cardiac echocardiography (TTE) unveiled circumferential fibrinous pericardial effusion, along with signs of right ventricular collapse, while the left ventricular ejection fraction remained within the normal range (Figure 3). The laboratory test results revealed significant indicators of severe hypothyroidism, including a thyroid-stimulating hormone (TSH) level of 14.45 µU/mL, low FT4 at 1.46 ng/dL, TSH receptor antibody levels were measured at 5.13 IU/L, and highly elevated anti-thyroid peroxidase antibodies at 31.74 IU/mL, all indicative of Hashimoto's thyroiditis. Renal function parameters, including creatinine at 0.59 mg/dL and urea concentration at 13 mg/dL, were within the normal range. There is no evidence of tuberculosis, cancer, or lupus in the patient. The sputum test of tuberculosis, antinuclear antibody test, and pathological anatomic measure were all negative. Additionally, cytological analysis of the pericardial fluid revealed an inflammatory response with no presence of malignant cells.

**Figure 4:**
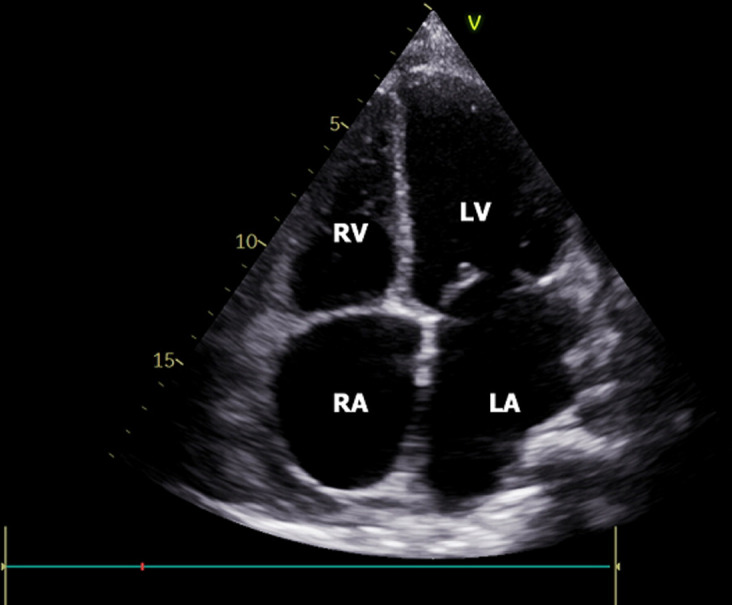
the follow-up echocardiography after pericardiocentesis

**Therapeutic intervention:** to alleviate the condition, a percutaneous pericardiocentesis procedure was carried out through subxiphoid access with a pericardiocentesis kit, guided by echocardiographic. Approximately 3000 ml of pale red fluid was successfully drained slowly at a rate of 500-1000 cc per day for five days, leading to improvement in the patient's clinical condition. The echocardiography follow-up after pericardiocentesis showed improvement in the right ventricle, and there was only a mild pericardial fluid (Figure 4). The patient was also given levothyroxine 75 μg tablets for her hypothyroidism therapy. A follow-up echocardiography six months later indicated the presence of a well-tolerated chronic pericardial effusion.

**Follow-up and outcomes:** the patient showed significant improvement after undergoing pericardiocentesis and receiving levothyroxine hormone therapy.

**Patient perspective:** the patient expressed immense satisfaction and gratitude, as after one week of hospital treatment, their initial severe shortness of breath complaint disappeared. Additionally, the pericardial fluid has significantly reduced, and the patient's thyroid hormone levels are now well-regulated with the prescribed medications.

**Informed consent:** a written informed consent was obtained from the patient for the publication of patient data and accompanying images.

## Discussion

Hashimoto's thyroiditis, an autoimmune thyroid disorder, often leads to gradual and nonspecific symptoms of hypothyroidism, which can go unnoticed for an extended time. Diagnosis may be delayed, and in rare cases, patients can experience life-threatening emergencies like cardiac tamponade. Although mild pericardial effusion is common in hypothyroidism, moderate to severe cases are rare, and cardiac tamponade due to pericardial effusion is extremely uncommon. Prompt recognition of hypothyroidism and the initiation of levothyroxine treatment are crucial for effective management [7].

Hypothyroidism affects various organ systems with varying manifestations depending on factors like its cause, duration, and extent. It primarily impacts the cardiovascular system, leading to bradycardia, diastolic hypertension, a narrow pulse pressure, and reduced pericardial activity. While ventricular arrhythmias are common, heart failure is rare due to the generally lowered cardiac output meeting reduced oxygen demands [8].

The incidence of pericardial effusion in hypothyroidism varies from 3% in mild cases to 80% in severe cases [3]. It results from increased capillary permeability, reduced lymphatic clearance, and enhanced salt and water retention. Cardiac tamponade, a rare complication of hypothyroidism, typically occurs in severe and long-standing cases. Although most patients seek medical attention before this stage, in rare instances, hypothyroidism can initially present with cardiac tamponade. Diagnosis is confirmed through echocardiography, and treatment involves pericardiocentesis and levothyroxine replacement therapy [9,10].

Electrocardiogram (ECG) findings can aid in diagnosis, with hypothyroidism commonly showing sinus bradycardia and prolonged QTc intervals. In contrast, cardiac tamponade often presents with sinus tachycardia, electrical alternans, and low-voltage QRS complexes. The presence of bradycardia or normal heart rate in cardiac tamponade suggests hypothyroidism as a potential cause. Chest radiogram showed cardiomegaly with globular enlargement of the cardiac silhouette with a “water bottle-shaped” configuration. Echocardiographic findings in cardiac tamponade include massive pericardial effusion and chamber collapse. Pericardial fluid in hypothyroidism is typically golden-colored, exudative with a high protein content, and predominantly contains lymphocytes [5]. Emergency pericardiocentesis is required for patients with cardiac tamponade, along with levothyroxine replacement therapy. Pericardial effusion typically resolves over 3 to 6 months with treatment [4].

## Conclusion

Severe hypothyroidism can cause pericardial effusion, but it is a rare cause of massive pericardial effusion leading to tamponade. Therefore, it is important to monitor the heart condition of patients with hypothyroidism continuously using echocardiography until they have euthyroid.
